# The Evaluation of Diagnostic Role of Cardiac Troponin T (cTnT) in Newborns with Heart Defects

**DOI:** 10.1100/2012/682538

**Published:** 2012-04-01

**Authors:** Agata Tarkowska, Wanda Furmaga-Jabłońska

**Affiliations:** Department of Neonate and Infant Pathology, Medical University of Lublin, 20095 Lublin, Poland

## Abstract

Heart diseases are a significant cause of morbidity and mortality in newborns. Diagnostic methods are often not sufficient or, in many cases, cannot be used. There is a great advance in medical knowledge concerning biomarkers in the diagnosis of circulatory system in adult patients. Among them, cardiac troponins play the main role. In current literature, there is not enough data concerning the possibility of using them in neonatal cardiac diagnostics. *Aim of the Study*. To evaluate diagnostic usefulness of cTnT in correlation with other markers of circulatory failure and myocardial damage in newborns with heart defects. *Patients and Methods*. The study involved 83 newborns up to 46 weeks of postmenstrual age. The exclusion criteria were severe perinatal asphyxia and presence of severe noncardiac diseases. Patients were divided into 2 main groups: group I—54 patients with congenital heart defects (CHDs), and group II (control)—29 healthy neonates. All patients underwent detailed examination of circulatory system. Cardiac troponin T (cTnT) concentrations were evaluated by Roche CARDIAC T Quantitive test. *Results*. Performed studies revealed that cTnT levels in newborns with heart pathology were significantly higher than in healthy ones. However, cTnT concentrations in patients with CHD did not correlate with clinical symptoms of heart failure, nor with echocardiographic markers of LV function. Type of heart defect did not influence cTnT levels as well. Only hemodynamic significance evaluated by echocardiography influenced the cTnT levels with statistical significance. *Conclusions*. (1) Statistically significant differences in cTnT levels between newborns with heart defects and healthy subjects were shown. (2) CTnT levels in newborns with heart defects refer only to hemodynamic significance of the defect.

## 1. Introduction

Heart diseases leading to circulatory failure are a significant cause of morbidity and mortality in newborns [1]. Diagnosis of early stages heart failure in newborn is difficult because clinical symptoms are nonspecific. The diagnostic methods indicating cardiac damage are often not sufficient or, in many cases, cannot be used due to high technical requirements or their invasive nature [2]. It is necessary to look for noninvasive markers that would enable wider diagnosis of heart muscle damage and cardiac insufficiency risk in neonates.

Cardiac troponins are protein components of the tropnin-tropomyosin complex in myocardium. Since troponins do not occur in extracellular space, their appearance in serum is sensitive and specific marker of myocardium damage [3]. Troponins appear in blood in 2 to 4 hours after insult, peak in about 12 h and then remain elevated for 7–10 days [3, 4].

Sensitivity of both cTnT and cTnI in the diagnosis of myocardial damage is clinically almost equal. They differ in intracellular compartments, biological half-life, and molecular weight [5]. There are also differences in the standardization and availability of commercial troponin kits. Absolute values of gained results are often incomparable, however, diagnostic features of particular methods are similar [6].

Cardiac troponins (cTn) are biochemical markers of myocardial injury with unquestionable significance in diagnostic strategy in adults [3, 7–11]. However, their role in diagnostics in neonates has not been fully explored yet. Cardiac troponins have not been used routinely in neonates because of insufficient data confirming their clinical utility in this age group. Studies conducted in other groups confirm the usefulness of troponins in clinical situations that lead to cardiomiocytes injury, including cardiac inflammatory diseases [2, 4, 5, 12–14]. In literature, the following applications of cTn in pediatrics are mentioned: acute myocarditis, heart arrhythmias, perinatal asphyxia in newborns, perioperative myocardial injury in patients operated for congenital heart diseases, drug-induced cardiotoxity, and cardiac transplantation [4, 5, 14].

## 2. The Aim of the Study

Was to evaluate the diagnostic usefulness of cTnT measurements in correlation with other markers of circulatory failure and myocardial damage in newborns with congenital heart defects.

## 3. Patients and Methods

The study involved 83 newborns up to 46 weeks of postmenstrual age. The gestational age of studied newborns was 25–42 weeks, mean: 38 weeks. Chronological age (in days from the date of birth) was 6–135 days, mean: 21 days. Postmenstrual age was 34–46 weeks, mean: 41 weeks. Birth weight of studied newborns was 585–5400 g, mean: 3137 g. The exclusion criteria were severe perinatal asphyxia (Apgar scale ≤ 4 points in 1st and 5th minute) and patients with signs of severe noncardiac diseases.

All patients underwent detailed subjective and physical examination. In patients with abnormalities in circulatory system echocardiography and electrocardiographic tests were performed. On the basis of performed examination, patients were divided into 2 main groups: group I—54 patients with congenital heart defects (CHDs), and group II (control)—29 healthy neonates up to 46 weeks of postmenstrual age. Newborns with CHD were divided into following subgroups: group Ia—with simple shunts and group Ib—with combined heart defects (Table 1). Patients in group I were also divided according to hemodynamic significance of the heart defect evaluated in echocardiography. Hemodynamic significance was evaluated on the base of echocardiographic parameters such as right ventricle and/or right atrium enlargement, accelerated blood flow in main pulmonary artery (MPA), and extension of MPA, presence of tricuspid regurgitation, extension of pulmonary veins. The particular criteria of hemodynamic significance depended on the type of heart defect. The following subgroups were stated: group Ii—with hemodynamic significant defect and group In—without hemodynamic significance. The comparison of clinical variables between particular groups is presented in Tables 4 and 5.

Each newborn with cardiac abnormality had the following examinations performed: echocardiography with left ventricle ejection fraction (EF LV) evaluation, chest X-ray, and blood pressure measurement. All patients were also evaluated with Ross'es heart failure in infants classification [15] and with Reithmann's pediatric heart failure score [16]. Basic laboratory tests were performed in each newborn. The study protocol was approved by the Ethics Committee of Medical University of Lublin.

Troponin *T* levels were evaluated in 150 *μ*L of whole blood by Roche CARDIAC *T* Quantitative test (third generation; Roche Diagnostics). The Roche CARDIAC *T* quantitive test includes two monoclonal antibodies specific for cardiac troponin *T*. Measuring range of the test is 0.03 to 2 ng/mL. The results are not influenced by hyperbilirubinemia (bilirubin <20 mg/dL), hemolysis nor lipemia (triglicerydes <440 mg/dL). Blood samples were collected into standardized heparinized tubes. The obtained results, after checking the normality of distribution, were statistically analyzed by the use of appropriate test with Statistica 9.0 packet. Right-handed asymmetry of certain distributions was eliminated by the use of logarithmic transformation. Dependency analysis was performed based on Pearson's linear correlation coefficient (r) or Spearman's (R) rank correlation test and *t*-test significance of the correlation coefficient in the population. The results were concerned as statistically significant when *P* < 0.05.

## 4. Results

**Figure 1:**
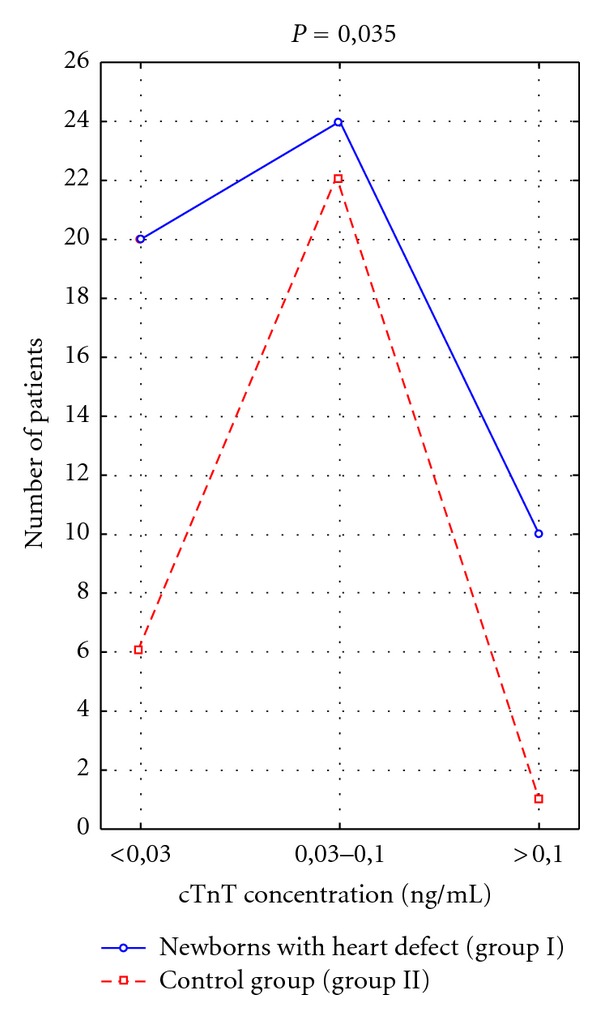
Number of patients with particular cTnT concentration ranges in studied groups.

Performed studies revealed that cTnT levels in newborns with heart pathology were significantly higher than in healthy ones (*P* = 0.035, Figure 1, Table 2). However, cTnT concentrations in patients with CHD did not correlate with clinical symptoms of heart failure evaluated with Ross'es scale (*R* = 0.095 and *P* = 0.493) as well as with Reithmann's classification (*R* = 0.076, *P* = 0.493). Cardiac TnT concentrations also did not correlate with echocardiographic markers of LV function, nor with the type of heart defect. The obtained results revealed that only hemodynamic significance of heart defect evaluated by echocardiography influenced the cTnT levels with statistical significance (*P* = 0.048, Table 3).

## 5. Discussion

Cardiac troponins are highly specific cardiac markers, extremely sensitive, and valuable in diagnostics of myocardial necrosis [17, 18]. Sobki et al. [19] in their paper demonstrated that troponins are very highly sensitive and specific for myocardial injury. Other studies showed that troponin elevations may occur in different cardiac pathologies, not only in ischemic heart disease [18]. According to studies conducted on adult patients, increased oxygen demand in myocardium and the increase of cardiac troponins serum concentration occur in clinical situations like: arrhythmias (atrial fibrillation, supraventricular tachycardia, etc.), in chronic and acute heart failure, or in myocarditis [17]. The accurate mechanisms responsible for the elevation of troponin serum concentration in diseases other than acute coronary syndromes remain in the course of research [18]. According to data from literature, elevated cTn concentrations in cardiac insufficiency are connected with the decrease of left ventricular ejection fraction and correlate with the severity of symptoms and with worse prognosis [17]. While these biomarkers were widely studied in adult patients, with structurally normal hearts, it is unknown if the results correlate with heart failure in newborns and infants with congenital heart defects. Typically, cardiac insufficiency in adults is usually connected with coronary disease, whereas in children heart failure is rather complication of structural abnormalities or primary myocardial dysfunction [20].

Shah et al. [20] in their study made an attempt to evaluate whether serum cTnI concentrations in children with functionally single-chamber heart may be used as biomarker of heart failure. The study included 29 children at the age of 1 month to 7 years, with functionally single-chamber heart. No differences in cTnI concentrations were found between patients with functionally one ventricle and heart failure and those without cardiac insufficiency. In most patients, cTnI concentrations were undetectable.

Similarly, on the basis of the results obtained in our study, no difference was stated between serum cTnT concentrations and clinical exponents of heart failure. The obtained results did not show correlation with clinical symptoms of heart failure evaluated with Ross'es heart failure in infants classification, nor with Reithmann's pediatric heart failure score. Heart failure is more frequently observed in neonates, especially in preterms [21]. In the available literature, there was no study evaluating correlation between clinical heart failure symptoms and cardiac troponins concentrations in newborns with congenital heart defects. The obtained results, similar as data from literature concerning older children with CHD, confirm that in opposite to adults, cardiac troponins concentration in pediatric patients does not correlate with the severity of clinical heart failure symptoms. Most probably, this follows different etiopathogenesis of heart failure in children and adults. Although, Muñiz [22] in his paper presented a case report of patient at the age of 9 weeks with combined congenital heart defect and chronic heart failure, in whom significantly elevated troponin levels were observed, however, it was only a single report. The author suggests that there is an increased risk of arrhythmia or heart function deterioration in infants with elevated heart enzymes.

At present, echocardiography is the best tool to evaluate heart structure and myocardial contractility [21]. According to data from literature, evaluating serum cardiac troponin concentrations as markers combined with results of echocardiography may largely facilitate making clinical decisions [21]. The available literature showed that cardiac troponins may also serve as useful complement in evaluation of respiratory distress syndrome and perinatal asphyxia in newborns [23]. In one of the studies, cTnT serum concentrations were correlated with echocardiographic measurements in preterm newborns in their 12th hour of life [24]. The authors showed significant negative correlation between cTnT and echocardiographic markers of myocardial function.

In our studies, no correlation between shortening fraction (SF) evaluated by echocardiography and cTnT concentration in newborns with CHD was observed. The above result is difficult to be referred with data from the literature, as yet only one study led by EL-Khuffash et al. [24] showed correlation between SF and cardiac troponins concentration in newborns. However, the mentioned study involved only preterms born in 26.1–29.5 weeks of gestational age, with structurally normal hearts, and troponin concentration evaluation was performed in the first day of life. Possibly, the noncompliance in our results and results of the cited study is due to differences between the groups of studied patients. On the other hand, SF is not a reliable indicator of left ventricle systolic function in newborns, because of the typical adaptation period high blood pressure in right ventricle, which influences septum movements [25]. In case of congenital structural heart defects, this discrepancy may be even more strongly expressed.

Structural heart defects are the most often inborn malformations diagnosed in the first year of life [21]. Studies conducted in adults with heart defects showed that mean cTnI concentration was higher in patients with aortic valve pathology compared with control group [26]. Also elevated pulmonary blood pressure might be connected with increased cTnI serum concentrations [26]. In available literature, there is a very small number of studies evaluating troponin concentrations in pediatric patients with heart defects. In order to determine the influence of congenital or acquired heart defects on cardiac troponin serum concentrations, Hirsch et al. [27] evaluated cTnI levels in two groups of children. Group A was represented by pediatric patients without diagnosed heart disease and stable patients with known congenital or acquired cardiac abnormalities. Group B was created from patients admitted to Intensive Care Unit: with normal ECHO results, with abnormal ECHO results, or after chest injury. The mentioned authors stated that cTnI levels are generally not elevated in children with stable heart disease or in children with systemic diseases.

On the basis of our studies, it was found that cTnT serum concentration is statistically significantly higher in newborns with CHD compared to patients from the control group. In available literature, it is difficult to find studies concerning troponin concentrations in neonatal patients with heart defects. One of the papers [23] showed that cTnT concentrations in newborns with persisted ductus arteriosus (PDA) significantly correlated with the arterial duct diameter, the shunt velocity, and end diastolic volume in descending aorta. The authors concluded that cTnT may be a useful marker of PDA significance and reaction to treatment, as it correlates with echocardigraphic PDA markers. Elevated cTnT concentration may reflect the potential myocardial damage caused by the presence of PDA. *Stealing* of oxygenated blood by arterial duct can influence the coronary flow, and potentially lead to ischemia [23].

In another research, conducted by EL-Khuffash et al. [24] on group of preterm newborns with PDA, cTnT evaluation, and ECHO examination were performed in the first day of life. Significant negative correlation was found between cTnT and echocardiographic markers of left ventricle (LV), including SF LV. Correlation between PDA diameter and cTnT was not confirmed. This was the only study as yet in which correlation between cTnT and EF LV in newborns was found. According to the authors, the above results may have practical application in indirect evaluation of myocardial function in newborns when echocdiography is not available. In the same paper, it was shown that cTnT did not depend on cardiac volume load.

In available literature, there were no other studies found concerning cardiac troponin concentrations in CHD in newborns before cardiosurgeric treatment. In our previous study, cTnI concentrations were evaluated in 41 newborns at the age of 7–28 days with CHD [28]. No differences were found in cTnI concentration in studied patients depending on type of heart defect (simple or combined). However, elevated cTnI levels were found in newborns with pulmonary hypertension (HP), secondary to structural heart defect compared to group without HP and the difference was close to statistical significance. Results of the present study does not confirm this observation, as no significant correlation was found between cTnT concentration and features of HP in newborns with CHD.

The results of our study confirmed that although cTnT concentrations are significantly higher in newborns with CHD compared to healthy ones, however, no significant difference was found depending on the type of the defect. In newborns with isolated left-to-right shunt defects, no significant correlation was observed between cTnT concentration and shunt velocity evaluated by echocardiography. Also, no significant correlation was found between cTnT concentration and the diameter of septal defect or PDA. However, the obtained results showed significant correlation between cTnT concentration and hemodynamic significance of heart defect evaluated on the basis of echocardiographic evaluation. Heart defects more hemodynamically significant carry greater risk of heart failure development and necessity to start appropriate treatment as soon as possible. The correlation between cTnT concentration and hemodynamic significance of CHD creates potential possibility for above biomarker to be used for early detection of newborns with significant heart defects, who need urgent cardiology consultation.

## 6. Conclusions

1. Statistically significant differences in cTnT levels between newborns with heart defects and healthy subjects were shown.
2. Cardiac TnT concentrations in newborns with CHD does nor correlate with clinical signs of heart failure nor with echocardigraphic markers of LV function.
3. Cardiac TnT concentrations in newborns with CHD does not depend on the type of the defect.
4. The statistically significant correlation was found between cTnT concentration and hemodynamic significance of CHD in examined newborns.

## Figures and Tables

**Table 1:** Characteristic of heart defects in group I patients.

Division of heart defects Group I (*N* = 54)		Type of defect	Number of patients
Hemodynamic significant defects (**Group Ii**) *N* = 29	Simple shunts (**Group Ia**) *N* = 16	ASD + VSD	10
		ASD + VSD + PDA	2
		ASD + PDA	2
		PDA	1
		ASD	1
	Combined heart defects (**Group Ib**) *N* = 13	CAVC	5
		FT4	2
		PS + ASD	1
		CoA + ASD	1
		TA	1
		L-TGA +VSD + PS	1
		DORV + ASD	1
		SA + PS + ASD + PDA	1
Defects without hemodynamic significance (**Group In**) *N* = 25	Simple shunts (**Group Ia**) *N* = 24	ASD	11
		ASD + PDA	7
		ASD + VSD	4
		ASD + VSD + PDA	2
	Combined heart defects (**Group Ib**) *N* = 1	PS + ASD	1

*N:* number of patients

ASD: atrial septum defect

VSD: ventricular septum defect

PDA: persistent ductus arteriosus

CoA: aortic coarctation

PS: pulmonary stenosis

DORV: double outlet left ventricle

TA: truncus arteriosus

TGA: transposition of great arteries

SA: aortic stenosis.

**Table 2:** Number of patients with particular cTnT concentration ranges in studied groups.

cTnT (ng/mL)	Newborns with heart defects group I (*N* = 54)	Control group group II (*N* = 29)	Statistical significance
**<**0,03	20	6	*P* = 0,035
0, 03–0, 1	24	22	
0, 1–2, 0	10	1	

*N*: number of patients.

**Table 3:** Troponin *T* concentration categories in dependence of heart defect hemodynamic significance.

	Hemodynamic significance group I (*N* = 54)		Statistical significance
TnT (ng/mL)	Significant (group Ii) *N* = 29	Not significant(group In) *N* = 25	
**<**0,03	7	13	*P* = 0,048
0,03–0,1	14	10	
0,1–2,0	8	2	

*N: *number of patients.

**Table 4:** Comparison of clinical signs of heart failure evaluated by the Rosse's heart failure in infants classification in studied groups.

Group		Class	Number of patients
I (*n* = 54)	Ii (*n* = 29)	I	22
		II	2
		III	2
		IV	3
	In (*n* = 25)	I	25
II (*n* = 49)		I	49

**Table 5:** Comparison of clinical signs of heart failure evaluated with the Reithmann's pediatric heart failure score.

Group		Score (points)	Number of patients
I (*n* = 54)	Ii (*n* = 29)	0–2	24
		3–6	2
		>6	3
	In (*n* = 25)	0–2	25
II (*n* = 49)		0–2	49
